# Spontaneous healing of an isolated posterior inferior cerebellar artery dissection without stroke: a case report

**DOI:** 10.1186/s12883-019-1352-0

**Published:** 2019-06-12

**Authors:** Yo Kishi

**Affiliations:** Oike Clinic, Kyoto, Japan

**Keywords:** Isolated posterior inferior cerebellar artery (PICA) dissection, Spontaneous healing, Headache, High-resolution MRI

## Abstract

**Background:**

Isolated posterior inferior cerebellar artery dissections can cause subarachnoid hemorrhages or infarctions. Surgical and endovascular treatments for hemorrhagic stroke cases and medical treatments using antithrombotic agents for ischemic stroke cases have been performed, but there are very few reports on nonstroke isolated posterior inferior cerebellar artery dissections, and the treatment strategy for nonstroke cases has not been established.

**Case presentation:**

A 48-year-old healthy male felt a severe, throbbing headache on the right side and came to our clinic on the fourth day following onset. MRI examinations revealed a right posterior inferior cerebellar artery dissection and showed no infarctions or hemorrhages. He was observed carefully with continuous monitoring of blood pressure, hydrated sufficiently, and given analgesic anti-inflammatory agents. Two weeks later, the dissected vessel’s diameter grew to the maximum size, though the patient’s headache rapidly improved around that day. Surgical or endovascular treatments for prevention of subarachnoid hemorrhage were recommended, but careful conservative therapy was continued in accordance with the patient’s wishes. Gradually, the dissection finding improved. Four months later, MRI examinations showed his right posterior inferior cerebellar artery was almost normal in size and shape.

**Conclusions:**

This is the first detailed report on a nonstroke isolated posterior inferior cerebellar artery dissection that spontaneously occurred and healed, observed by serial high-resolution MRI examinations.

**Electronic supplementary material:**

The online version of this article (10.1186/s12883-019-1352-0) contains supplementary material, which is available to authorized users.

## Background

Isolated posterior inferior cerebellar artery dissections (iPICADs) can cause subarachnoid hemorrhages or infarctions [1]. The incidence of iPICAD had been thought to be very rare. The reports of iPICAD with stroke, however, are increasing with the advancement of high-resolution MRI techniques. Surgical and endovascular treatments for hemorrhagic stroke cases and medical treatments using antiplatelet or anticoagulant agents for ischemic stroke cases have been reported. The treatment strategy for iPICADs without stroke, however, is not yet established because the reports on nonstroke iPICADs are still rare. Herein, a nonstroke iPICAD case that spontaneously healed is reported.

## Case presentation

**Fig. 1:**
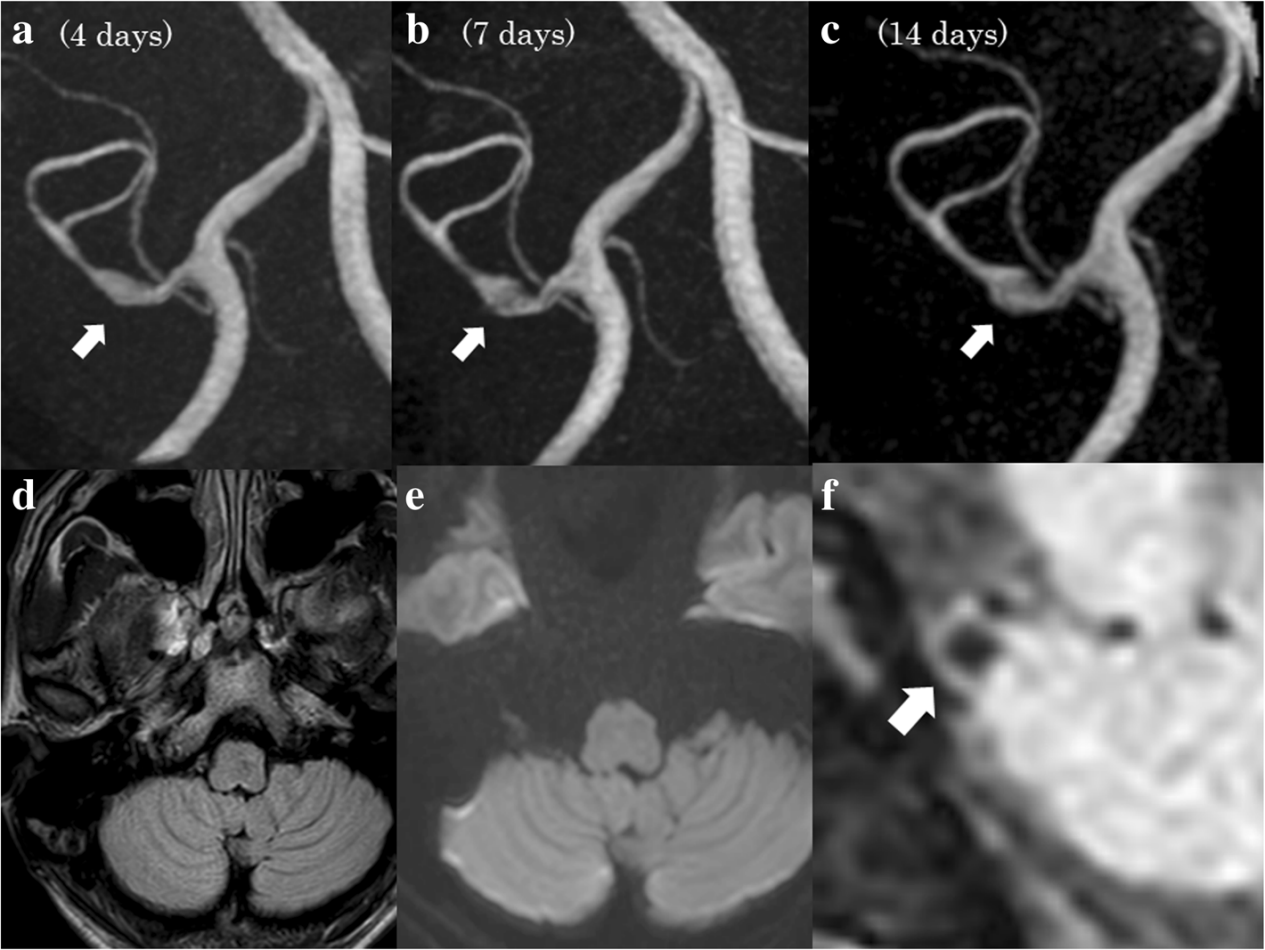
**a**, **b** and **c** are MRA (MIPs). A (4 days after onset) shows ‘pearl and string sign’ like finding of the patient’s right PICA. The PICA inner diameters are growing gradually (7 days later) (**b**) and (14 days later) (**c**). The FLAIR and DWI shows no hemorrhages and infarctions, respectively (**d** and **e**). T1-weighted vessel wall image depicts no apparent intramural hematomas of the patient’s right PICA (F). (All arrows indicate the affected PICAs)

**Fig. 2:**
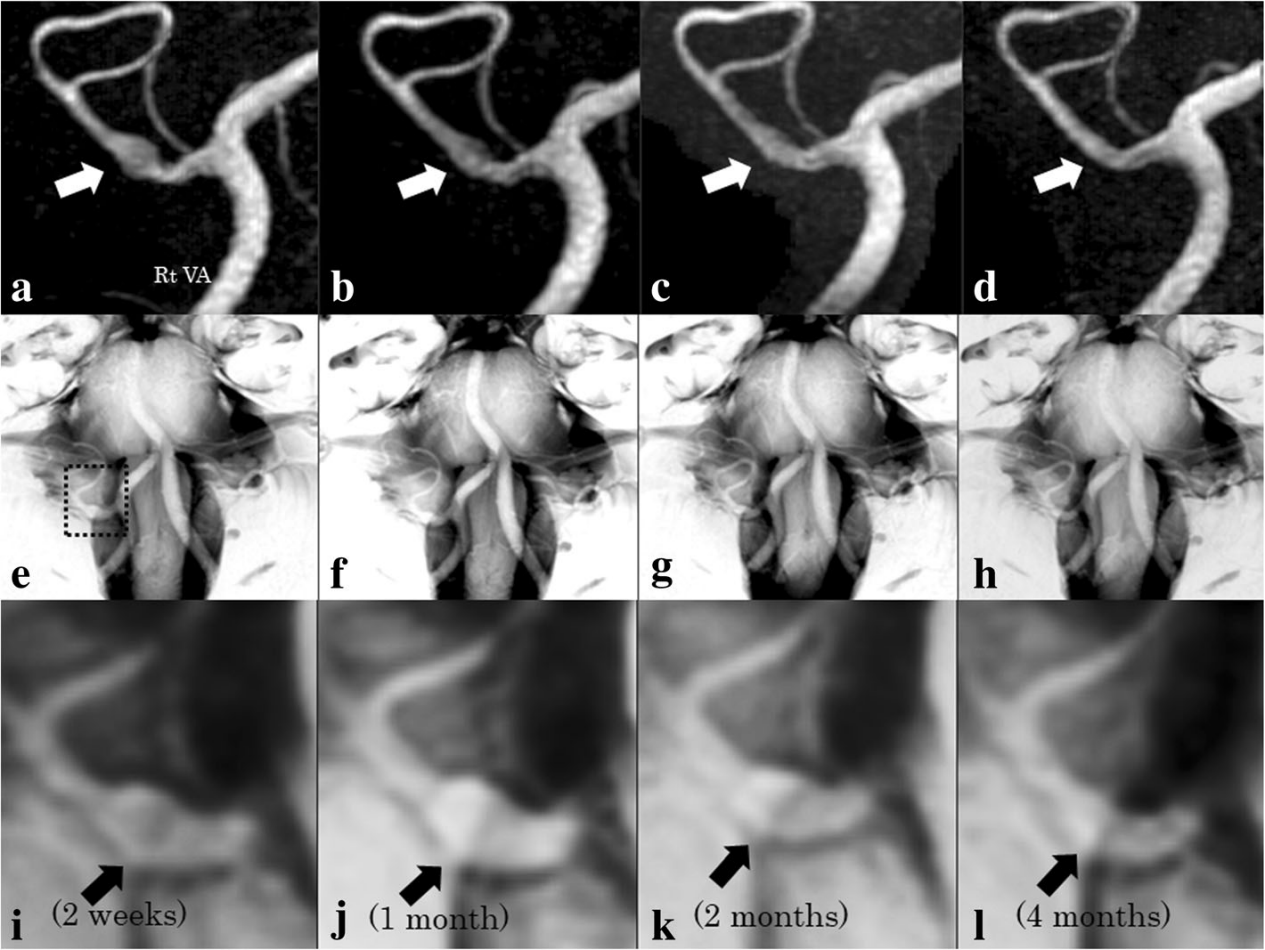
MRA (MIP) in upper raw (**a**-**d**), BPAS in middle raw (**e**-**h**) and BPAS zooming in the affected PICA in lower raw (**i**-**l**). From the left column to the right column, the data of 2 weeks after the onset (**a**, **e** and **i**), 1 month (**b**, **f** and **j**), 2 months (**c**, **g** and **k**) and 4 months (**d**, **h** and **l**) are lined. Chronological changes of PICA inner diameter (MIP) and outer diameter (BPAS) are shown. MIPs show that the inner diameter of the right PICA grows maximally on the 14th day and are gradually downsizing. These MRI findings show the inner diameter reduction precedes the outer diameter’s shrinkage

**Fig. 3:**
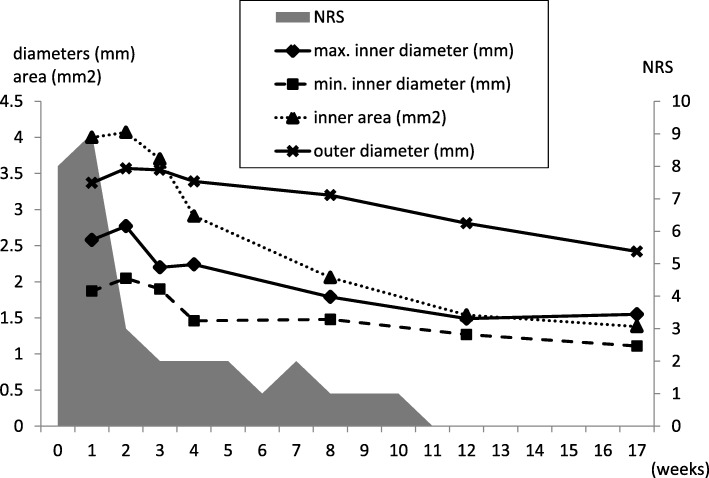
The patient’ headache severity was measured by numerical rating scale (NRS) every day. The inner diameter of the affected PICA was measured using cross-sectional view of T1-weighted HRVWI and the outer diameter was measured using original data of T2-weighted HRVWI (BPAS). Two weeks later after the onset of headache, the dissected vessel’s diameter grew up to the maximal, though NRS score of the patient’s headache improved steeply on the eighth day. Since then, NRS scores had been kept within 2. The maximum inner and outer diameters shrunk gradually to normal size

A 48-year-old male, who had no past medical or traumatic history and no family history of cerebral artery dissections, suddenly felt a severe, throbbing headache on the right side and came to our clinic on the fourth day following onset. MRI examinations showed a tiny dissection-like finding (pearl and string sign-like) on his right proximal segment (tonsillomedullar segment) of posterior inferior cerebellar artery (PICA) (Fig. 1a). No intramural hematoma, double lumen finding or intimal flap were observed, but PICA dissection (PICAD) could not be ruled out, and the patient was therefore carefully observed, with continuous monitoring of blood pressure, heart rate and other vital signs; also, he was hydrated sufficiently and given analgesic anti-inflammatory agents. MRA on the seventh day revealed that there was an association between the change in shape and volume of the PICA and the time elapsed, strongly suggesting PICAD (Fig. 1b). The other MRI sequences showed no infarctions or hemorrhages (Fig. 1d, e. Conventional angiography was not performed because the PICA could be observed by serial high-resolution MRI examinations and angiography was not considered necessary in this case. The inner and outer diameters of the dissected PICA were measured by MRI T1-weighted high-resolution vessel wall imaging (HRVWI) (Fig. 1f) and T2-weighted HRVWI (basi-parallel anatomical scanning (BPAS); Fig. 2), respectively. The severity of the headache was assessed by Numerical Rating Scale (NRS) every day. Two weeks after the onset of headache, the diameter of the dissected vessel grew to the maximum size (Fig. 2a), though the patient’s headache improved markedly on the eighth day (Fig. 3). At that time, surgical or endovascular treatment to prevent subarachnoid hemorrhage was recommended; however, the patient, who had been relieved of severe headache, desired to continue conservative therapy. Four weeks after the onset, the dissection finding on MIP images began to improve. Eight weeks after the onset, his PICA looked almost normal on MIP and T1-weighted HRVWI, though the outer diameter was still bulging on a T2-weighted HRVWI (BPAS). Finally, four months after the onset, the outer diameter was observed to be almost normal in size and shape (Fig. 2). The patient has resumed activities, such as marathon racing, again.

## Discussion and Conclusions

MRI examinations showed no apparent intramural hematoma, double lumen, or intimal flap indicating artery dissection. PICA is a tiny vessel and it is possible that these findings could not be detected even by high-resolution MRI techniques. Recently, the chronological changes in the size and shape of the affected vessels have been considered to be one of the criteria for diagnosing cervicocephalic artery dissections, including intracranial small vessels such as PICA. Table 1 shows the Spontaneous Cervicocephalic Arterial Dissections Study (SCADS) criteria. According to the criteria, the patient was diagnosed with PICAD (criteria 5, 7, and 8 matched) [2].

**Table 1:** Diagnostic criteria for cervicocephalic arterial dissection

Major criteria	
1. “Double lumen” or “intimal flap” demonstrated on either DSA, MRI, MRA, CTA, or duplex ultrasonography 2. “Pearl and string sign” or “string sign” demonstrated on DSA 3. Pathological confirmation of arterial dissection	
Minor criteria	
4. “Pearl sign” or “tapered occlusion” demonstrated on DSA 5. “Pearl and string sign,” “string sign,” or “tapered occlusion” demonstrated on MRA 6. “Hyperintense intramural signal” (corresponding to intramural hematoma) demonstrated on T1-weighted MRI	
Additional criteria	
7. Change in arterial shape demonstrated on either DSA, MRI, MRA, CTA, or duplex ultrasonography 8. No other causes of arterial abnormalities	
*Definite dissection*	
Presence of one or more major criteria, or presence of one or more minor criteria and both of 2 additional criteria	
*Probable dissection*	
Presence of one or more minor criteria	

Conventional angiography may have provided useful information to confirm the patient’s PICA condition; however, for this patient, serial high-resolution MRI examinations, including HRVWI, were sufficient to confirm the condition of the vessel. T1-weighted HRVWI was used to confirm the intraluminal size change. The outer diameter was followed by basi-parallel anatomical scanning (BPAS), one type of T2-weighted HRVWI [3].

Among cervicocephalic artery dissections, vertebral artery dissections (VADs) are common, and the therapeutic strategy for VAD has been established [4]. The reports of iPICAD with stroke are increasing with the advancement of high-resolution MRI techniques, but those without stroke are still limited. In the last decade, over 70 cases of iPICAD have been found in the literature, but almost all the cases included subarachnoid hemorrhages [5] or infarctions [6]. There have been only two nonstroke iPICAD cases reported, and one of these was diagnosed with coexisting VAD [7]. As mentioned above, PICAD is often a tiny finding, even with high-resolution MRI techniques, and can therefore be overlooked unless accompanied by a symptomatic stroke. The patient in the present case was fortunately suspected to have iPICAD at the beginning of the clinical course and was followed using high-resolution MRI examinations, though he presented only with headache.

It is interesting that the severity of the patient’s headache might be related to the chronological changes of the MRI findings. The patient felt a severe, throbbing headache continuously while MRI findings of PICAD were worsening. After the occipital pain was relieved, his PICAD finding stopped worsening and gradually improved on MRI examinations.

Conservative therapy is usually selected for nonstroke VAD, whereas the therapeutic strategy for nonstroke iPICAD is not yet determined. The other case of the reported two iPICAD without stroke was surgically treated to prevent subarachnoid hemorrhage because the dissection finding by radiological examination was progressive [8]. It might be permissible to perform any surgical and/or endovascular treatments in such a case. However, as in the present case, it is also possible that nonstroke iPICAD may heal spontaneously.

This is the first detailed report on a nonstroke iPICAD that spontaneously occurred and healed, observed by MRI examinations. In this case, serial high-resolution MRI examinations clearly showed the spontaneous healing course of iPICAD without stroke.

## Additional files

Additional file 1: **Figure S4.** The inner diameters and areas of the dissected PICA were measured using cross-sectional view of T1-weighted HRVWI. (TIF 744 kb)

Additional file 2: **Figure S5.** The outer diameters of the dissected PICA were measured using original data of T2-weighted HRVWI (BPAS). The measured values are shown in Fig. 3. (TIF 744 kb) (TIF 603 kb)

## Data Availability

All data generated or analyzed during this study are included in this published article and its Additional files 1 and 2.

## Abbreviations

BPAS: Basi-parallel anatomical scanning
CTA: Computed tomographic angiography
DSA: Digital subtraction angiography
DWI: Diffusion weighted image
FLAIR: Fluid-attenuated inversion recovery
HRVWI: High-resolution vessel wall imaging
iPICAD: Isolated posterior inferior cerebellar artery dissection
MIP: Maximum intensity projection
MRA: Magnetic resonance arteriogram
MRI: Magnetic resonance imaging
NRS: Numerical rating scale
VAD: Vertebral artery dissection
