# Supplementary figures and images for: Spontaneous healing of an isolated posterior inferior cerebellar artery dissection without stroke: a case report
Source: BMC Neurol. 2019 Jun 12;19:124. doi: 10.1186/s12883-019-1352-0 (PMC6560718; doi:10.1186/s12883-019-1352-0)

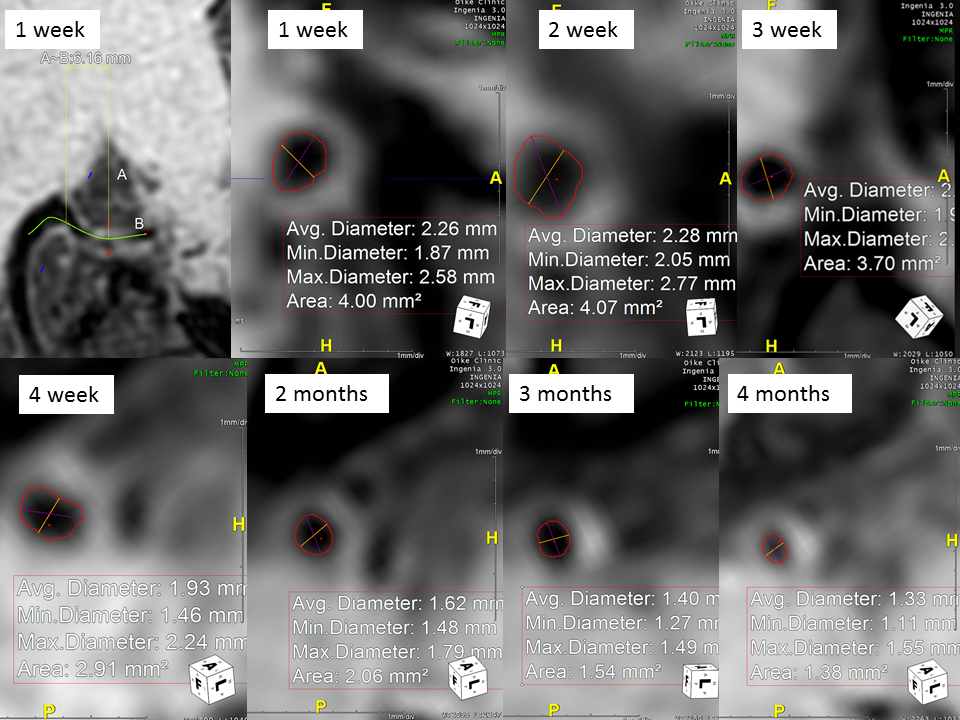

Supplement: Supplementary file 1 — Figure S4. The inner diameters and areas of the dissected PICA were measured using cross-sectional view of T1-weighted HRVWI. (TIF 744 kb) [file 12883_2019_1352_MOESM1_ESM.tif]

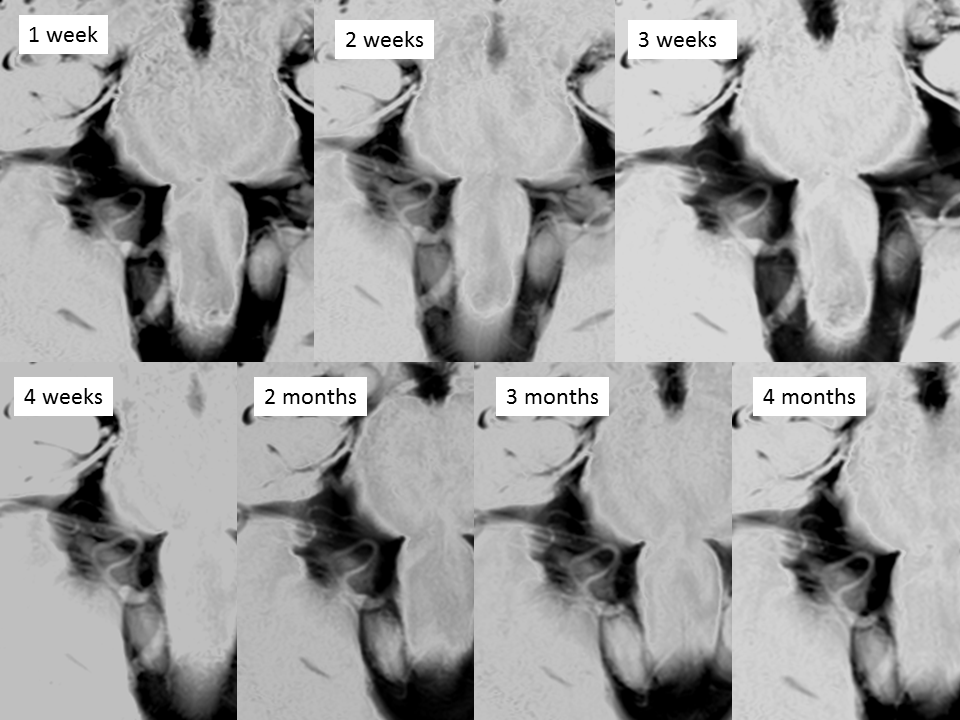

Supplement: Supplementary file 2 — Figure S5. The outer diameters of the dissected PICA were measured using original data of T2-weighted HRVWI (BPAS). The measured values are shown in Fig. 3. (TIF 744 kb) (TIF 603 kb) [file 12883_2019_1352_MOESM2_ESM.tif]
